# Expression of Eosinophilic Subtype Markers in Patients with Kawasaki Disease

**DOI:** 10.3390/ijms231710093

**Published:** 2022-09-03

**Authors:** Ling-Sai Chang, Kuang-Den Chen, Ying-Hsien Huang, Ho-Chang Kuo

**Affiliations:** 1 Kawasaki Disease Center, Department of Pediatrics, Kaohsiung Chang Gung Memorial Hospital, Chang Gung University College of Medicine, Kaohsiung 33302, Taiwan; 2 Institute for Translational Research in Biomedicine, Liver Transplantation Center, Department of Surgery, Kaohsiung Chang Gung Memorial Hospital, Chang Gung University College of Medicine, Kaohsiung 33302, Taiwan; 3 Department of Respiratory Therapy, Kaohsiung Chang Gung Memorial Hospital, Kaohsiung 833, Taiwan

**Keywords:** allergic diseases, coronary artery lesions, eosinophils, IVIG, Kawasaki disease

## Abstract

Purpose: Eosinophils may rise to a higher level in the acute phase of Kawasaki disease (KD) both before and after intravenous immunoglobulin (IVIG) therapy. A substantial body of research was carried out on the association between KD and allergic diseases. Eosinophils play an important role in type 2 inflammation. Recent studies have shown that there are two distinct subtypes of eosinophils. In addition to their role in inflammation, lung-resident eosinophils (rEOS) also regulate homeostasis. Inflammatory eosinophils (iEOS) reflect type 2 inflammation in tissues. iEOS were considered the primary eosinophils in non-severe allergic asthma, while rEOS were thought to be the primary eosinophils in severe non-allergic eosinophilic asthma. This case–control study aimed to investigate the marker expression of eosinophilic subtypes in KD patients. Materials and Methods: The marker expressions of eosinophilic subtypes in the leukocytes of patients with KD were evaluated by the recently established KDmarkers online tool, a web server including gene expression data. Finally, the results were validated with a quantitative reverse transcriptase polymerase chain reaction (RT-PCR). We analyzed the mRNA expression levels of *SELL* and *IL10RA* in leukocytes from KD patients and febrile children. Results: Included in our screening tools were transcriptome arrays, which provided clues showing the importance of rEOS, whose role was identified by three genes (lower *IL10RA*, higher *SELL,* and *SERPINB1* than controls). In contrast, the iEOS representative gene *CD101* was not elevated in KD. It was found that the gene *IL10RA*, a marker of inflammatory eosinophilic leukocytes, was more highly expressed in the leukocytes of KD patients (*n* = 43) than febrile controls (*n* = 32), especially those without coronary artery lesions (CAL) (*n* = 26). Before treatment, *SELL* expression was higher in leukocytes of CAL patients (CAL, 1.33 ± 0.18, *n* = 39; non-CAL, 0.87 ± 0.12, *n* = 55; *p* = 0.012). *SELL* was significantly higher after half a year compared to febrile controls. Conclusions: To our knowledge, this is the first study to demonstrate that KD patients have increased *SELL* than febrile controls after 6 months of treatment. We present evidence here that dynamically different eosinophilic involvement exists between KD patients with and without CAL. The role of eosinophilic subtypes in KD patients warrants further investigation.

## 1. Background

Discovered over 60 years ago, Kawasaki disease (KD) has since been one of the major acquired heart diseases with rare life-threatening complications [1]. It is thus an important medical issue to track the long-term health of children with KD, a typically self-limited condition. Studies have found that children with KD are more likely to be hospitalized for infections or allergic diseases [2,3,4]. In addition to cardiovascular sequelae, KD has been shown to have other long-term effects, leading to common health problems such as allergic diseases and compromised immunity [5,6]. Our latest study of immunoglobulin (Ig)M receptors is the first to show that *FCMR* transcripts decline after six months of KD [6]. The National Health Insurance Research Database shows that KD patients are more likely to develop allergic diseases, such as asthma and rhinitis, after recovery than the control group [7]. Increases in eosinophil cationic protein (ECP), interleukin (IL)-5, eosinophils, and total IgE in children with KD were considered markers of allergy [8,9]. The nasal epithelium is the main entry point into the airways. At mucosal sites, plasma cells produce IgA dimers that neutralize pathogens and particles. A recent study has shown that IgA levels have been related to coronary artery lesions (CAL) in KD [10]. Eosinophils are required to generate and maintain mucosal IgA plasma cells [11]. Eosinophils play important roles in type 2 inflammation, host immune defense against infection, and various biological functions including beneficial effects, immune regulation, and homeostasis [12,13,14]. Early studies showed that eosinophil levels were highly elevated in the acute phase of KD before and after intravenous immunoglobulin (IVIG) therapy [9]. Tsai et al. demonstrated that eosinophils > 1.5% may be helpful in diagnosing KD in other febrile patients [15]. Liu’s findings suggest that eosinophilia may be an important motivating factor in the diagnosis of KD [16]. The highest percentage of eosinophils in the convalescent phase after IVIG has long been recognized [17]. Eosinophilia (≥4%) after IVIG treatment was detected as a predictor of IVIG responsiveness and the Z-score of eosinophils was detected as a predictor of CAL [9,18]. Eosinophils were found to accumulate in epicardial microangiopathy in the autopsy of four KD patients [19].

Recent findings highlighted that multiple functions of eosinophils may be attributed to the subpopulation [20,21]. Marked phenotypic heterogeneity included surface markers, anatomical location, response to IL-5 and nuclear morphology. Transcriptional profiling of mouse eosinophils identified distinct gene signatures. However, subpopulations of eosinophils were largely unknown in human diseases. Lung-resident eosinophils (rEOS) in the steady-state normal lung also regulate homeostasis, termed homeostatic eosinophils, Siglec-F^int^CD62L^+^CD101^low^ cells with a ring-shaped nucleus [20,22,23]. The predominant eosinophil in non-severe allergic asthma is inflammatory (iEOS), whereas the predominant eosinophil in severe non-allergic eosinophilic asthma is the rEOS [24]. iEOS recruited after being challenged with house dust mites in mice are IL-5-dependent, defined as SiglecF^high^CD62L^−^CD101^high^ cells with segmented nuclei [23]. Transcriptomic profiles showed the alpha subunit of the *IL10RA*-encoding IL-10 receptor as a significantly and differentially expressed gene between iEOS and rEOS [20]. Compared with sibling controls, children with KD and without CAL developed allergic rhinitis and any other exacerbated forms of allergy. This finding is consistent with the previously observed lower T helper (Th)2 cytokines in KD patients with CAL [8]. Whether pro-inflammatory receptor P2 and anti-inflammatory receptor P1 behave differently has not been resolved [13]. Matucci et al. found a high proportion of inflammatory eosinophils in patients with severe asthma, especially in nasal polyps [25].

The final product of rEOS is L-selectin encoded by *SELL*, also known as CD62L in which the recovery phase of KD tends to be higher than the acute or subacute phase [26]. Plasma L-selectin levels tend to be lower in patients with CAL than in patients without CAL. This finding is consistent with decreased eosinophils in CAL patients. Eosinophilic chronic sinusitis (ECRS) is a type of rhinitis characterized by prominent eosinophilic infiltration. L-selectin plays a role in eosinophil recruitment in ECRS human nasal mucosa [27]. However, there is little ongoing research or available information on the eosinophilic subtypes of KD. This case–control study was designed to examine whether the expression of different markers of eosinophils is altered in children with KD.

## 2. Materials and Methods

**Figure 1:**
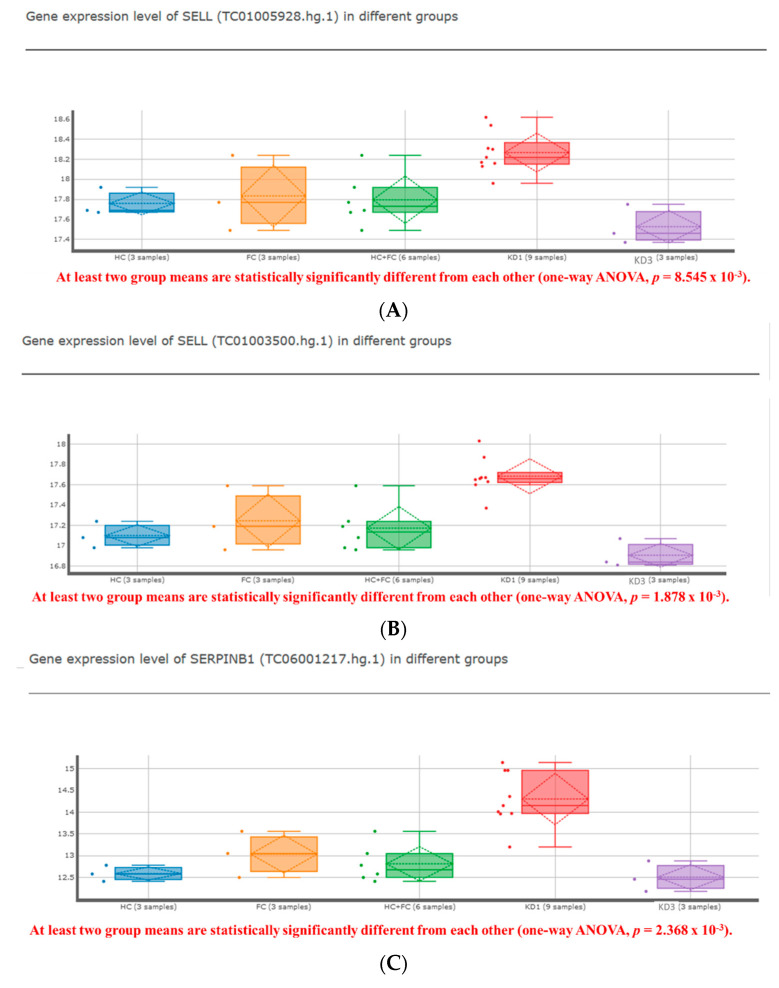

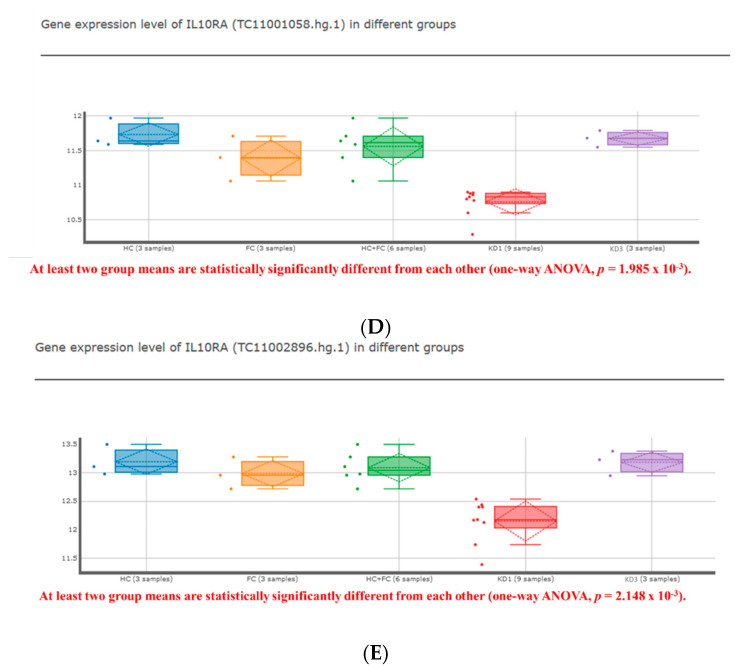
Expression analysis of (**A**,**B**) *SELL*, (**C**) *SERPINB1,* and (**D**,**E**). *IL10RA* in leukocytes assessed using KDmarkers tool. The representative gene *SELL* of lung-resident eosinophils was significantly higher in Kawasaki disease. ANOVA, analyses of variance; FC, febrile control; HC, non-febrile control; KD1, pre-IVIG (intravenous immunoglobulin) patients with Kawasaki disease (KD); KD3, KD patients three weeks after IVIG.

mRNA expression profiles were obtained from leukocyte samples collected during a previously published experiment and uploaded to the NCBI GEO database (GSE109351 series); all methods and materials used to obtain data are provided in the cited publications from a total of 50 KD patients before IVIG and 18 KD patients three weeks after IVIG, while 18 non-febrile and 18 febrile controls were included in Human Transcriptome Array analysis (Table 1) [6,28,29]. Each sample was pooled from six patients (Figure 1). Marker expression of eosinophilic subtypes in leukocytes of KD patients was assessed using the recently established KDmarkers online tool (https://cosbi.ee.ncku.edu.tw/KDmarkers/, accessed on 13 January 2022), a web server that contains gene expression data of the GeneChip Human Transcriptome Array 2.0 (HTA 2.0, Affymetrix, Santa Clara, CA, USA) covering more than 285,000 full-length transcripts [29]. In order to identify the significant ratio of transcriptome level and 1/ratio (fold changes), one-way analysis of variance was conducted by the default setting of the commercial microarray tool Partek. Finally, the results were checked and proven by quantitative reverse transcriptase polymerase chain reaction (RT-PCR).

### mRNA Detected by the Quantitative Reverse Transcriptase-Polymerase Chain Reaction

The study employed a retrospective case–control study in a single center. We used RT-PCR to validate the mRNA levels of *SELL* and *IL10RA* in KD patients and controls. All patients with fever plus ≧4 of 5 clinical criteria (extremity changes, rash, conjunctivitis, oral changes, and cervical lymphadenopathy) or fever plus fewer principal criteria and coronary artery abnormalities identified by echocardiography met the diagnostic criteria for KD specified by the American Heart Association after excluding other febrile illnesses [30]. This study was approved by the Institutional Review Board (IRB) of Kaohsiung Chang Gung Memorial Hospital (202002142A3) and carried out according to the Declaration of Helsinki. After a full description of the study, all parents or guardians signed informed consent. All KD patients were admitted to the pediatric ward of Chang Gung Memorial Hospital in Kaohsiung, Taiwan and received a single high-dose IVIG of 2 g/kg each time. The first venous blood sample was collected from each patient on the first day of diagnosis or before IVIG therapy (KD1). Samples were obtained 3 to 7 days and 3 weeks post-treatment (KD2/KD3) following acute and subacute IVIG treatment. KD4 was defined as six months after IVIG. We measured the lumen diameter of the coronary arteries using two-dimensional echocardiography. CAL was defined as coronary findings in KD patients meeting the following criteria: ≥3.0 mm in inner diameter in children under 5 years old, ≥4.0 mm in inner diameter in children over 5 years old, a diameter ≥1.5 times greater than that of an adjacent segment, and irregular luminal contour according to the guidelines of the Japanese Ministry of Health or a z-score of ≥2.5 [31,32]. Patients whose symptoms did not meet the KD criteria were excluded. Age- and sex-matched febrile patients with no history of KD were studied as controls for comparison. The fever-controlled patients were admitted to the hospital with acute infection.

Genes validated in leukocytes were selected to be associated with rEOS (*SELL*) and iEOS (*IL10RA*). Sequence information for all primers used were: *SELL*, FP—CAGTCTACCTGCAGCACAGC; RP—TGGGTGCTCTGACATTTC; *IL10RA*, FP—CCCTGTCCTATGACCTTACCG; RP—CACACTGCCAACTGTCAGAGT; 18s, FP—GTAACCCGTTGAACCCCATT; RP—CCATCCAATCGGTAGTAGCG. Final data were normalized using the geometric mean of housekeeping genes, 18s. Total RNA from peripheral leukocytes was extracted from cell lysates using a miRNA isolation kit (Life Technologies, Carlsbad, CA, USA) according to the manufacturer’s instructions. An equal amount of 1 µg of RNA was then reverse-transcribed into cDNA using the High-Capacity cDNA Reverse Transcription Kit (Thermo, Waltham, MA, USA). RT-PCR was performed in triplicate on a 7500 Fast Real-Time PCR System (Appliedbiosystems, Waltham, MA, USA) using Fast SYBR Green PCR Master Mix (Appliedbiosystems, Thermo, Waltham, MA, USA). Relative amounts of all mRNAs were normalized to 18s mRNA expression levels using the 2^−ΔΔCT^ method; results are shown as fold changes relative to controls.

Continuous data in results, tables, and figures are presented as mean and standard error. We evaluated quantitative data between controls and KD or between KD patients with CAL and without CAL with the Mann–Whitney *U* test. We performed the necessary chi-square or Fisher’s exact test on categorical data. We applied all statistical analyses using SPSS version 12.0 for Windows (IBM, New York, USA), and we considered two-sided tests with a *p*-value of < 0.05 to be of statistical significance.

## 3. Results

Representative datasets that have been detailed with fold changes and *p*-values are summarized in Table 2. We found that the representative gene *SELL* for rEOS was significantly higher in KD (Figure 1) [20]. In the different mRNA expressions of rEOS and iEOS in the transcriptome arrays below, we set the cutoff criteria to be all *p*-values < 0.01 in KD before IVIG compared to non-febrile controls, febrile controls and three weeks after IVIG [20].

rEOS, *PDE2A, LAIR1, SELL, PRG3, RUNX3, P2RX4, IL12RB2, METRNL, PON2, NEDD4, ANXA1, LDLR, MYC, S1PR2, SERPINB1, Rnase1, Rnase3, Rnase12* (Table 2A).

iEOS, *CD34, CD101, RIPK2, HCAR2, ITGA1, ITGAX, TLR4, BCL2A1, IL13RA1, IL1RN, SLC3A2, TREM1, C3AR1, H1F0, CD300C (AF251705), MIF, OLFM4, GRN, LPIN1, CD33, IL10RA, RETN, ACP5, CXCR2, IL6, ADORA2A, BCL6, PGF* (Table 2B).

Figure 1A–E show that the KDmarkers online tool (https://cosbi.ee.ncku.edu.tw/KDmarkers/, accessed on 13 January 2022) identified three genes (lower *IL10RA*, higher *SELL*, and *SERPINB1*) and indicated the role of rEOS after filtering by the cut-off criteria we set. In contrast, the iEOS representative gene *CD101* was not elevated in KD (Table 2B).

To sum up the salient features of the analysis, several findings are worth noting. In this study, we examined the mRNA expression of the above 28 iEOS markers in healthy controls (HC), febrile controls (FC), acute KD (KD1), and KD patients (KD3) 21 days after IVIG treatment (Figure 1). It was found that, among the 28 iEOS markers examined, 9 markers (namely *HCAR2, ITGAX, TLR4, C3AR1, OLFM4, GRN, CD33, RETN,* and *BCL6*) had significantly higher mRNA expression in the acute phase of KD compared with HC (Table 2). IVIG treatment resulted in a significant reduction in the facet of 12 genes, including *HCAR2, ITGA1, ITGAX, TLR4, IL1RN, C3AR1, OLFM4, GRN, CD33, RETN, CXCR2,* and *BCL6*. The expressions of *LPIN1* and *IL10RA* were lower in the acute phase of KD and were significantly increased after IVIG treatment.

We also examined the mRNA expression of 18 rEOS markers in KD patients and controls (Table 1). Of the 18 rEOS markers observed, 6 markers (*LAIR1, SELL, NEDD4, LDLR, SERPINB1,* and *Rnase3*) significantly increased mRNA expression in acute phase KD and then significantly decreased after IVIG treatment. One marker, *RUNX3*, decreased mRNA expression in the acute phase of KD and then increased after IVIG treatment. *SELL* between KD and controls was significantly different in the transcriptome array (Figure 1). The results after IVIG showed that *SELL* was significantly lower than that of KD patients in the pre-IVIG group.

**Figure 2:**
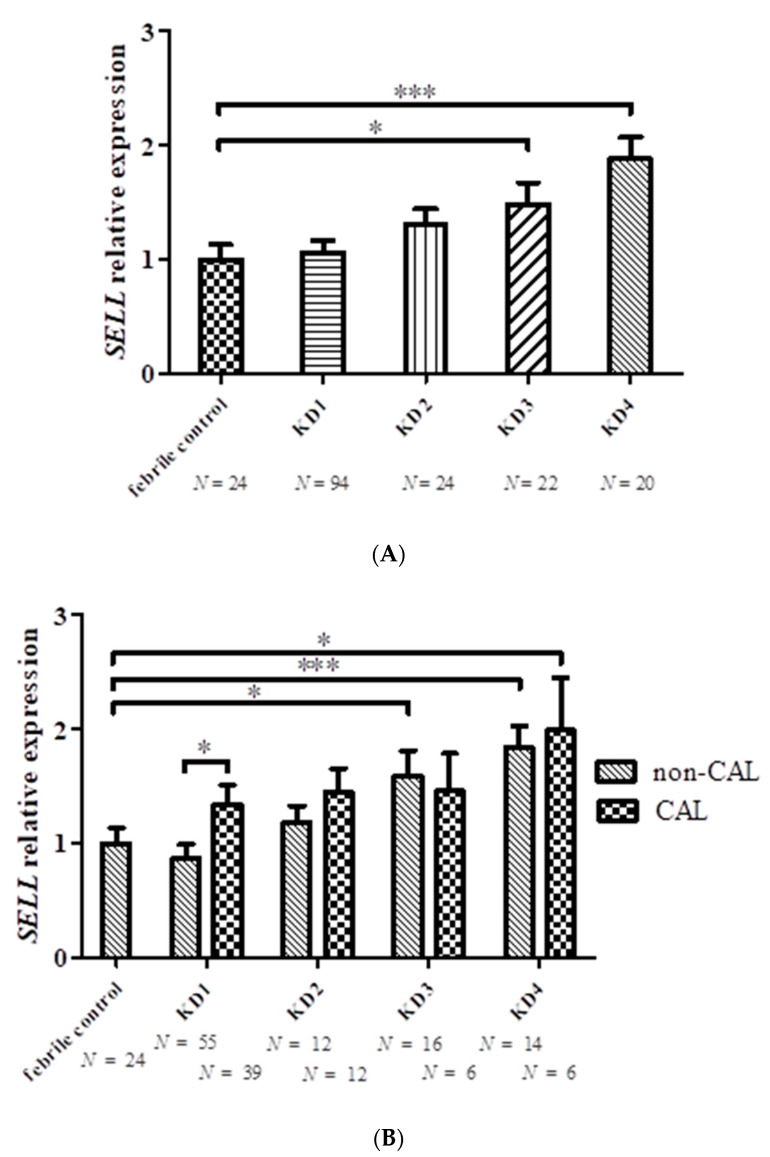
*SELL* mRNA levels (**A**) in febrile controls and patients with Kawasaki disease (KD1, before treatment; KD2, 3–7 days after treatment; KD3, three weeks after treatment; KD4, six months after treatment); (**B**) in KD patients with and without coronary artery lesions (CAL). * denotes significance (* *p* < 0.05; *** *p* < 0.001) between controls and KD patients, and KD patients with and without CAL.

**Figure 3:**
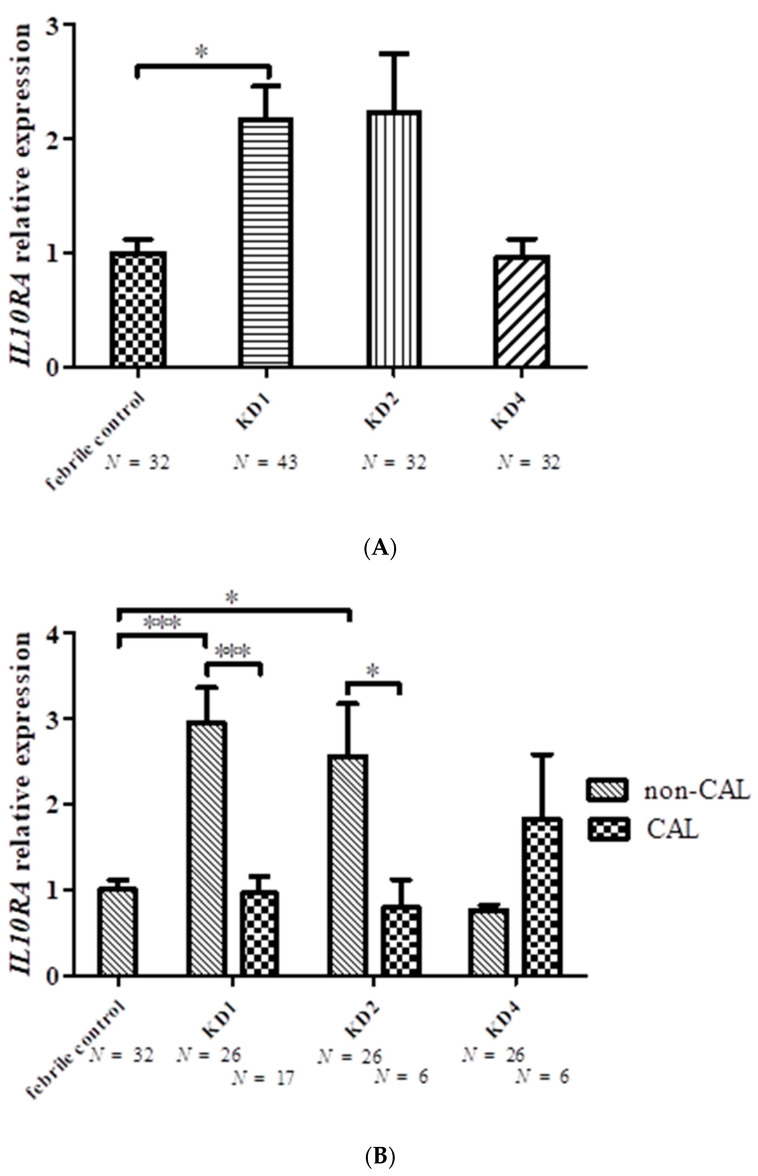
*IL10RA* mRNA levels (**A**) in febrile controls and patients with Kawasaki disease (KD1, before treatment; KD2, 3-7 days after treatment; KD4, six months after treatment); (**B**) in KD patients with and without coronary artery lesions (CAL). * denotes significance (* *p* < 0.05, *** *p* < 0.001) between controls and KD patients, and KD patients with and without CAL.

The results of the array analysis were verified by RT-PCR. *SELL* mRNA levels were significantly elevated in leukocytes of KD3 (*n* = 22) and KD4 (*n* = 20) patients compared with febrile controls (*n* = 24) (Figure 2 and Table 3). Patients with CAL (*n* = 39) had elevated *SELL* levels compared with patients without CAL before treatment (*n* = 55) (CAL, 1.33 ± 0.18; non-CAL, 0.87 ± 0.12; *p* = 0.012). *SELL* mRNA levels increased significantly in patients without CAL after three weeks and six months compared with controls (see Figure 2). In addition, *IL10RA* was used for further validation by RT-PCR. However, we found similar *IL10RA* mRNA levels in the controls and KD patients after 6 months of treatment (see Figure 3 and Table 4). It was observed that levels of *IL10RA* in leukocytes increased significantly in KD1 (*n* = 43, compared with febrile controls (*n* = 32) (KD1, 2.17 ± 0.30; fever control, 1 ± 0.12; *p* = 0.013). The *IL10RA* mRNA expression levels were significantly different between patients with and without CAL before and after IVIG (*p* < 0.001 and = 0.043, respectively).

## 4. Discussion

To our knowledge, this is the first study to explore the expression of eosinophil subtype markers in KD and the first to track them during the course of KD-related disease. It is critical to determine whether eosinophils are involved during KD. Research on the role of eosinophils will guide new strategies for allergic intervention after KD. We present evidence here that dynamically different eosinophilic involvement exists between KD patients with and without CAL.

The protein produced by the representative gene *CD101* of iEOS is immunoglobulin superfamily member 2. As both classically activated macrophages and markers of alternately activated macrophages show increased mRNA expression during the acute phase of KD, iEOS is also important in KD [33]. Yoon et al. performed experimental studies and showed iEOS with an expression of Toll-like receptor *(TLR) 4*, and *IL10R* regulates inflammatory and anti-inflammatory polarization of macrophages, which is very similar to the behavior of macrophages in acute KD [34]. The decrease in *IL10RA* mRNA after IVIG is consistent with previous reports of decreased ECP after IVIG [8,25]. The results of increased *IL10RA* between KD and controls as written here support previous findings on diagnostic biomarkers for KD [16]. The enhancement of IgE-mediated mast cell activation by IL-10 is critical for the development of food allergy in mouse models [35]. Lee et al. found significantly elevated IL-10 in KD patients [36]. The alpha subunit of the IL-10 receptor (encoded in the *IL10RA* gene) is expressed on hematopoietic cells such as T, B, NK, mast, dendritic cells, and eosinophils. Interleukin-10 receptor expression and signaling are downregulated in CD4+ T cells from lupus nephritis patients with higher clinical scores [37]. Eosinophils can promote autoantibody-driven inflammatory processes in anti-neutrophil cytoplasmic antibody-associated vasculitis by increasing the surface expression of activating Fc receptors [38].

RT-PCR validation of *SELL* showed no difference in *SELL* between febrile controls, which is in line with the literature showing no difference in CD62L expression on polymorphonuclear leukocytes between KD patients and normal subjects [39]. However, single-cell RNA sequencing identified expanded SELL+CD14+CD16- monocytes, which were poorly differentiated and associated with neutrophil activation in KD [40]. The discrepancy between *SELL*’s gene expression array and RT-PCR validation results may be due to the increased number of cases. Among other important and distinct rEOS genes in KD, *SERPINB1*, which encodes SerpinB1 belonging to the serine protease inhibitor family, functions as a signature gene for maintaining neutrophil survival and Th17 cells [41]. The elevation of *SERPINB1* is consistent with a Th17/Treg imbalance in KD [42]. SerpinB1 is involved in inhibiting acute oxidative stress and attenuating endothelial cell apoptosis [43]. L-selectin is the smallest angioselectin encoded by *SELL* and is also known as CD62L. Recent evidence suggests that L-selectin overexpression in multiple tumor-infiltrating immune cells mitigates tumor growth and correlates with favorable survival outcomes in breast cancer [44]. The expression of *SELL* and *SERPINE1* in KD demonstrates the role of rEOS in regulating immunity overactivated by KD immunity.

Among the many topics to be explored in future research, some important ones can be listed as follows. Prospective cohort studies that allow multiple testing are required to characterize the subtype properties of eosinophils and their possible roles in the pathogenesis of inflammation or anti-inflammatory effects against KD. Future work will hopefully clarify the important concern about the histological proof of eosinophils in situ by mouse models [45]. Neutralizing antibodies directed against IL-5 (α–IL-5) treatment did not impact the numbers of rEOS but substantially inhibited the recruitment of iEOS in the mice’s lungs. Anti-IL5 for KD patients with an iEOS phenotype or in KD patients with prognostic biomarkers of future allergic diseases would be considered [46]. An area of future research that should be considered is prognostic and therapeutic biomarkers of long-term allergic complications, the influence on acute treatment, and the strategy for preventing them.

## 5. Concluding Remarks

The conclusions drawn from the observations were mainly based on the following investigations: KD patients without CAL had lower *SELL* mRNA levels than patients with CAL, and KD patients with lower *IL10RA* mRNA levels were more likely to develop CAL. Evidence from various sources suggests that eosinophilic subtypes may play a role in KD. The eosinophilic subtype is far from being functionally understood and characterized. The assessment of how inflammatory responses are modulated may contribute to the interpretation and confirmation of eosinophil subtypes associated with KD.

## Figures and Tables

**Table 1:** Demographic characteristics of the subjects for establishing KDmarkers online tool.

	Non-Febrile Controls	Febrile Controls	KD before IVIG	KD after IVIG
	(*n* = 18)	(*n* = 18)	(*n* = 50)	(*n* =1 8)
Age (years)	2.46 ± 0.35	1.97 ± 0.26	1.36 ± 0.14	1.90 ± 0.32
Male/female ratio	1	0.8	1.17	1

IVIG, intravenous immunoglobulin; KD, Kawasaki disease.

**Table 2:** (**A**) Molecular biomarker of lung-resident (Table 2A) and inflammatory (Table 2B) eosinophils for Kawasaki disease by transcripts expressions; (**B**) Transcript expressions of inflammatory eosinophils between Kawasaki disease patients and control subjects.

(**A**)								
**Gene Symbol**	**RefSeq**	**Column ID**	**Fold-Change** **(KD1 vs. HC)**	***p*** **-Value** **(KD1 vs. HC)**	**Fold-Change** **(KD1 vs. FC)**	***p*** **-Value** **(KD1 vs. FC)**	**Fold-Change** **(KD3 vs. KD1)**	***p*** **-Value** **(KD3 vs. KD1)**
*PDE2A*	NM_001143839	TC11002059.hg.1	1.133	0.223	1.074	0.474	1.040	0.743
*LAIR1*	NM_002287	TC19001844.hg.1	1.386	0.001 **	1.130	0.108	−1.421	<0.001 **
*SELL*	NM_000655	TC01003500.hg.1	1.355	0.005 **	1.312	0.009 **	−1.484	0.001 **
*PRG3*	NM_006093	TC11001790.hg.1	1.035	0.718	−1.046	0.634	1.032	0.739
*RUNX3*	NM_001031680	TC01002370.hg.1	−1.274	0.004 **	−1.224	0.011 *	1.245	0.007 **
*P2RX4*	NM_002560	TC12000956.hg.1	1.010	0.864	−1.148	0.033 *	1.025	0.656
*IL12RB2*	NM_001559	TC01000741.hg.1	−1.046	0.537	−1.247	0.013 *	−1.116	0.154
*MATERNAL*	NM_001004431	TC17000959.hg.1	−1.026	0.715	1.024	0.739	1.019	0.788
*PON2*	NM_000305	TC07001617.hg.1	−1.076	0.395	−1.081	0.369	1.057	0.519
*NEDD4*	NM_006154	TC15001471.hg.1	1.452	0.031 *	1.424	0.038 *	−1.441	0.033 *
*ANXA1*	NM_000700	TC09000335.hg.1	1.415	0.092	1.709	0.018*	−1.525	0.049 *
*LDLR*	NM_000527	TC19000191.hg.1	1.322	0.017 *	1.016	0.870	−1.418	0.005*
*MYC*	NM_002467	TC08000749.hg.1	−1.061	0.334	−1.191	0.016 *	1.116	0.093
*S1PR2*	NM_004230	TC19001160.hg.1	−1.099	0.489	−1.218	0.168	1.117	0.417
*SERPINB1*	NM_030666	TC06001217.hg.1	1.955	0.001 **	1.736	0.004 **	−2.036	<0.001 **
*Rnase1*	NM_002933	TC14000902.hg.1	1.088	0.367	−1.099	0.316	−1.046	0.624
*Rnase3*	NM_002935	TC14000072.hg.1	2.016	0.002 *	1.475	0.039 *	−1.836	0.005
*Rnase12*	NM_001024822	TC14000890.hg.1	−1.008	0.854	−1.045	0.310	1.014	0.745
(**B**)								
**Gene Symbol**	**RefSeq**	**Column ID**	**Fold-Change** **(KD1 vs. HC)**	***p*** **-Value** **(KD1 vs. HC)**	**Fold-Change** **(KD1 vs. FC)**	***p*** **-Value** **(KD1 vs. FC)**	**Fold−Change** **(KD3 vs. KD1)**	***p*** **-Value** **(KD3 vs. KD1)**
*CD34*	NM_001025109	TC01003777.hg.1	1.005	0.949	−1.010	0.905	1.052	0.537
*CD101*	NM_001256106	TC01001025.hg.1	1.183	0.097	1.006	0.946	−1.202	0.074
*RIPK2*	NM_003821	TC08000545.hg.1	−1.010	0.961	1.523	0.071	−1.056	0.793
*HCAR2*	NM_177551	TC12002078.hg.1	2.185	0.025 *	1.328	0.345	−2.924	0.005 **
*ITGA1*	NM_181501	TC05003439.hg.1	1.100	0.334	1.083	0.413	−1.283	0.027 *
*ITGAX*	NM_000887	TC16000375.hg.1	1.774	0.009 **	1.578	0.026 *	−1.712	0.012 *
*TLR4*	NM_003266	TC09000601.hg.1	1.858	0.019 *	1.658	0.044*	−2.145	0.007 **
*BCL2A1*	NM_001114735	TC15001719.hg.1	1.242	0.144	1.615	0.007 **	−1.348	0.057
*IL13RA1*	NM_001560	TC0X000575.hg.1	1.294	0.185	1.380	0.108	−1.426	0.081
*IL1RN*	NM_173843	TC02000720.hg.1	1.465	0.054	1.222	0.271	−1.657	0.018 *
*SLC3A2*	NM_001012662	TC11000567.hg.1	−1.160	0.057	−1.281	0.006 **	1.182	0.036 *
*TREM1*	NM_001242589	TC06001717.hg.1	−1.027	0.888	1.250	0.252	−1.047	0.807
*C3AR1*	NM_004054	TC12001171.hg.1	3.061	0.003 **	1.172	0.577	−2.732	0.006 **
*H1F0*	NM_005318	TC22000288.hg.1	1.103	0.293	−1.055	0.555	−1.089	0.354
*CD300C (AF251705)*	NM_006678	TC17001858.hg.1	1.205	0.176	−1.079	0.562	−1.195	0.193
*MIF*	NM_002415	TC22001456.hg.1	−1.119	0.402	−1.182	0.225	1.018	0.892
*OLFM4*	NM_006418	TC13000228.hg.1	2.604	0.023 *	1.253	0.527	−2.573	0.024 *
*GRN*	NM_002087	TC17000570.hg.1	1.515	0.041 *	−1.057	0.752	−1.662	0.018 *
*LPIN1*	NM_145693	TC02000078.hg.1	−1.082	0.037 *	−1.095	0.021 *	1.077	0.046 *
*CD33*	NM_001772	TC19000775.hg.1	1.197	0.012 *	1.042	0.480	−1.167	0.024 *
*IL10RA*	NM_001558	TC11001058.hg.1	−1.351	<0.001 **	−1.262	0.002 **	1.300	0.001 **
*RETN*	NM_020415	TC19000133.hg.1	1.365	0.042 *	−1.004	0.974	−1.453	0.020 *
*ACP5*	NM_001111034	TC19001188.hg.1	−1.023	0.731	−1.111	0.133	1.007	0.914
*CXCR2*	NM_001168298	TC02001292.hg.1	1.531	0.066	1.306	0.219	−1.894	0.013 *
*IL6*	NM_000600	TC07000137.hg.1	−1.071	0.337	1.077	0.296	−1.004	0.960
*ADORA2A*	NM_000675	TC22001457.hg.1	−1.025	0.658	−1.053	0.375	1.121	0.069
*BCL6*	NM_001130845	TC03002096.hg.1	2.047	0.005 **	1.675	0.024 *	−2.072	0.004 **
*PGF*	NM_001207012	TC14001313.hg.1	−1.007	0.951	−1.018	0.882	1.094	0.465

KD1: Kawasaki disease before IVIG treatment; KD3: Kawasaki disease > 3 weeks after IVIG treatment; FC: febrile control; HC: non-febrile control. * indicates *p* < 0.05 and ** indicates *p* < 0.01 between the groups.

**Table 3:** (**A**) Demographic characteristics of the subjects with Kawasaki disease (KD) for *SELL* mRNA; (**B**) *SELL* mRNA levels between patients with and without coronary artery lesions (CAL).

(**A**)								
	**Febrile Controls**			**KD**			***p*-Value**	
	**(*n* = 24)**			**(*n* = 94)**				
Age (years)	2.14 ± 0.26			1.84 ± 0.17			0.088	
male	10			60			0.042	
female	14			34				
(**B**)								
		Age (years)		*SELL*				
KD1		CAL(−)	CAL(+)	*p* Value	CAL(−)	CAL(+)	*p* Value	
		(*n* = 55)	(*n* = 39)		(*n* = 55)	(*n* = 39)		
		1.89 ± 0.19	1.78 ± 0.31	0.118	0.87 ± 0.12	1.33 ± 0.18	0.012 *	
KD2		CAL(−)	CAL(+)	*p* Value				
		(*n* = 12)	(*n* = 12)					
		1.06 ± 0.15	0.94 ± 0.13	0.525	1.18 ± 0.15	1.45 ± 0.20	0.326	
KD3		CAL(−)	CAL(+)	*p* Value				
		(*n* = 16)	(*n* = 6)					
		1.74 ± 0.33	1.20 ± 0.23	0.417	1.59 ± 0.23	1.46 ± 0.33	0.883	
KD4		CAL(−)	CAL(+)	*p* Value				
		(*n* = 14)	(*n* = 6)					
		2.53± 0.38	1.55 ± 0.18	0.070	1.84 ± 0.19	1.99 ± 0.46	1.000	

(* *p* < 0.05).

**Table 4:** (**A**) Demographic characteristics of the subjects with Kawasaki disease (KD) for *IL10RA* mRNA; (**B**) B *IL10RA* mRNA levels between patients with and without coronary artery lesions (CAL).

(**A**)						
	**Febrile Controls**		**KD**			***p* Value**
	**(*n* = 32)**		**(*n* = 43)**			
Age (years)	2.53 ± 0.24		2.16 ± 0.29			0.055
male	20		34			0.114
female	12		9			
(**B**)						
	Age (years)		*IL10RA*			
KD1	CAL(−)	CAL(+)	*p*-Value	CAL(−)	CAL(+)	*p*-Value
	(*n* = 26)	(*n* = 17)		(*n* = 26)	(*n* = 17)	
	2.16 ± 0.33	2.17 ± 0.54	0.535	2.96 ± 0.4	0.96 ± 0.19	<0.001 *
KD2	CAL(−)	CAL(+)	*p*-Value			
	(*n* = 26)	(*n* = 6)				
	1.97 ±0.30	1.32 ± 0.36	0.530	2.56 ± 0.62	0.79 ± 0.32	0.043 *
KD4	CAL(−)	CAL(+)	*p*-Value			
	(*n* = 24)	(*n* = 6)				
	2.41± 0.29	2.30 ± 0.54	0.876	0.77 ± 0.61	1.83 ± 0.75	0.464

(* *p* < 0.05).

## Data Availability

The data that support the findings of this study are available on request from the corresponding author.

## Abbreviations

CAL, coronary artery lesions; ECP, eosinophil cationic protein; iEOS, inflammatory eosinophils; IgE, immunoglobulin; IL, interleukin; IVIG, intravenous immunoglobulin; KD, Kawasaki disease; LTB4, Leukotriene B4; M1, classically activated macrophages; M2, alternatively activated macrophages; rEOS, lung-resident eosinophils; RT-PCR, quantitative reverse transcriptase-polymerase chain reaction; SELL, Selectin L.
